# Association of TNF-α, IGF-1, and IGFBP-1 levels with the severity of osteopenia in mice with nonalcoholic fatty liver disease

**DOI:** 10.1186/s13018-023-04385-1

**Published:** 2023-12-01

**Authors:** Tong-Hao Wang, Jian-Biao Li, Yong-Gang Tian, Jin-Xin Zheng, Xiao-Dong Li, Shu-zhang Guo

**Affiliations:** 1 Department of Orthopedics, The Third Central Hospital of Tianjin; The Third Central Clinical College of Tianjin Medical University; Tianjin Key Laboratory of Extracorporeal Life Support for Critical Diseases; Artificial Cell Engineering Technology Research Center, Tianjin; Tianjin Institute of Hepatobiliary Disease, 83 Jintang Road, Hedong District, Tianjin, 300170 China; 2 Department of Hepatobiliary Surgery, The Third Central Hospital of Tianjin; Tianjin Key Laboratory of Extracorporeal Life Support for Critical Diseases; Artificial Cell Engineering Technology Research Center, Tianjin; Tianjin Institute of Hepatobiliary Disease, Tianjin, 300170 China

**Keywords:** Nonalcoholic fatty liver disease, Osteoporosis, Tumor Necrosis factor-alpha, Insulin-Like growth factor I, Insulin-Like growth factor binding protein-1

## Abstract

**Backgrounds:**

Nonalcoholic fatty liver disease (NAFLD) exhibits a close association with osteoporosis. This work aims to assess the potential effects of NAFLD on the progression of osteopenia in animal models.

**Methods:**

Forty-eight C57BL/6 female mice were randomly divided to wild-type (WT) group and high-fat diet (HFD) group. The corresponding detections were performed after sacrifice at 16, 24 and 32 weeks, respectively.

**Results:**

At 16 weeks, an remarkable increase in body weight and lipid aggregation in the hepatocytes of HFD group was observed compared to the WT group, while the bone structure parameters showed no significant difference. At 24 weeks, the levels of TNF-α and IL-6 in NAFLD mice were significantly increased, while the level of osteoprotegerin mRNA in bone tissue was decreased, and the level of receptor activator of nuclear factor Kappa-B ligand mRNA was increased. Meanwhile, the function of osteoclasts was increased, and the bone microstructure parameters showed significant changes. At 32 weeks, in the HFD mice, the mRNA levels of insulin-like growth factor-1 (IGF-1), runt-related transcription factor 2, and osterix mRNA were reduced, while the insulin-like growth factor binding protein-1 (IGFBP-1) level was increased. Simultaneously, the osteoblast function was decreased, and the differences of bone structure parameters were more significant, showing obvious osteoporosis.

**Conclusions:**

The bone loss in HFD mice is pronounced as NAFLD progresses, and the changes of the TNF-α, IL-6, IGF-1, and IGFBP-1 levels may play critical roles at the different stages of NAFLD in HFD.

## Main Points

- Bone loss in the HFD mice becomes increasingly apparent with NAFLD progression.
- The changes of the TNF-α, IL-6, IGF-1, and IGFBP-1 levels are important at the different stages of NAFLD in the HFD mice.

## Introduction

As one of the most common chronic metabolic liver disorders, nonalcoholic fatty liver disease (NAFLD) is a clinicopathological syndrome characterized by excessive deposition of fat in hepatocytes, including simple fatty liver disease (SFL), nonalcoholic steatohepatitis (NASH), and associated cirrhosis. Multiple factors contribute to the pathogenesis of NAFLD, such as insulin resistance (IR) and overweight. In a recent epidemiological study, NAFLD is confirmed to be related to osteoporosis [1], sharing common risk factors, including aging, inappropriate lifestyles and sex hormone deficiency, indicating that they might be related in some way [2].

With several publications supporting the correlation between a reduced bone mineral density (BMD) and NAFLD [3, 4]. While it is conflict to use overweight and IR to explain this phenomenon. Recently, whether there is a positive correlation between bodyweight and bone mass has been discussed in obese-related studies [5]. Overweight and the increased body mass index (BMI) are related to bone mass because the increase in weight and fat during exercise cause bones to withstand greater forces and promotes an increase in bone density [6]. Another study found that IR was associated with the increased BMD, while this increase did not affect the serum cytokine levels [7]. Therefore, the changes of bone microstructure and BMD in NAFLD remain intensely debated.

NAFLD is a multifactorial disease that is thought to be caused by multiple genetic and environmental factors. The pathogenesis of NAFLD is explained by the ‘2-hit’ hypothesis [8], and the ‘first hit’ was considered to participate in accumulation of triglyceride (TG) in hepatocytes or steatosis. It is reported that the ‘second hit’ is closely linked to oxidative stress and inflammatory cytokines. The accumulation of IR and TG, chronic inflammatory processes, vitamin D deficiency, and insulin-like growth factor-1 (IGF-1) reduction play important roles in the pathogenesis of NAFLD, which may be related to osteoporosis.

Herein, we aims to analyze the changes in bone microarchitecture and mineralization over time by microcomputed tomography (micro-CT) in a mouse model of NAFLD induced by a high-fat diet (HFD) and hereby address the following aims: (1) investigate the changes of bone microstructure and pathogenesis in the progression of NAFLD; (2) investigate the role of TNF-α and IGF-1 during osteoporosis in NAFLD mice.

## Materials and methods

### Animals

Forty-eight 6-week-old wild-type C57BL/6 female mice (weight, 20 ± 2 g) were purchased from Vital River Laboratories (Beijing, China). All the mice were housed in a suitable environment with a temperature of 20–22 °C, humidity of 50–60% in light/dark cycle for 12 h/12 h. Mice were free to eat and drink, and the bodyweight was measured during the study period. The animal experimental procedures were approved by the Experimental Animal Committee at The Third Central Hospital of Tianjin. All the experiments performed in this study were approved and conducted according to standard guidelines of the Institutional animal ethical committee (2020–142). Following adaptation for 1 week. The mice were randomly divided into two groups: wild-type C57BL/6 mice (WT group, *n* = 24); wild-type C57BL/6 mice with HFD (HFD group, *n* = 24). HFD (D12492, Research Diet, USA) is made up of protein (20 kcal%), carbohydrates (20 kcal%), and fat (60 kcal%). At 16 weeks, 24 weeks and 32 weeks, eight mice in each group were anaesthetized with sodium pentobarbital (50 mg/kg, intraperitoneal injection) and decapitated. Blood, liver, femur and tibiae were collected and stored for later use.

### Measurement of body and organ weight

The weight of mice was measured at the end of 16, 24 and 32 weeks. After the mice were sacrificed, the liver of the mice was extracted, fully washed by normal saline, drained and then, weighed with an electronic balance. Liver index were calculated using the liver weight and the equivalent body weight of respective animals.

### Biochemical marker analysis

After the mice were sacrificed, blood samples were collected from the eyeballs, and after static coagulation for 15–20 min, serum was separated by rotating at 4000 rpm for 10 min and stored at − 20 °C. Blood biochemical markers such as Alanine transaminase (ALT), serum glucose (GLU), aspartate aminotransferase (AST), triglycerides (TG) levels, and total cholesterol (TC) were detected by a Hitachi automatic biochemical analyzer (HITACHI, Japan). In addition, the IL-6 and TNF-α levels in serum were measured by enzyme-linked immunosorbent assay (ELISA) kits produced by Shanghai Fusheng Industrial Co., Ltd. (Shanghai, China). The test procedures were executed according to the manufacturers’ instructions. Serum GLU and insulin were analyzed using a glucometer (Bayer, Germany) and ELISA kit (Roche, Switzerland), respectively. Serum IGF-1 and insulin-like growth factor binding protein-1(IGFBP-1) were determined using a radioimmunoassay kit (ALPCO, Windham, NH) and ELISA kit (Insight Genomics, Falls Church, VA), respectively. Total serum 25-hydroxyvitamin D (25(OH)D) level was measured using liquid chromatography mass spectroscopy (LC–MS).

### Liver and bone histological analysis

To investigate liver steatosis and bone structure changes, liver sections and the right tibiae sections from the same part of each animal were fixed in 4% paraformaldehyde for 24 h, dehydrated with alcohol gradient, embedded in paraffin wax, and stained with hematoxylin and eosin (HE) for pathological examination. The histological results showed that the adipose area of liver tissue was more than 1/3, indicating that the NAFLD animal model was successfully established.

### Micro-CT detection of bone microstructure

The right femur of mice was subsequently isolated and immobilized at 4 °C PBF (pH 7.4) for 24 h. The 70% ethanol was used to dehydrate each sample for 48 h and then, removed into a 8 mL tube filled with 70% ethanol to measure bone microarchitecture and BMD using micro-CT (Skyscan 1076 Micro-CT System; Skyscan, Aartselaar, Belgium). All scans were performed using X-ray energy at 50 kV and 200 μA, and the samples were scanned over one entire 360° rotation, with an exposure time of 2000 ms/frame. An isotopic resolution of 9.0 μm × 9.0 μm × 9.0 μm voxel size was selected to reveal the microstructure of the mouse trabecula. The incremental angle around the sample was set as 0.5° to obtain 1000 two-dimensional images. Mimics 17.0 (Materialize Company, Belgium) software was used for 3D image reconstruction and analysis. The epiphyseal trabeculae and cortical bones were selected as areas of interest for analysis. BMD, trabecular separation (Tb.Sp), bone volume ratio (BV/TV), trabecular thickness (Tb.Th), trabecular number (Tb.N), connectivity density (Conn.D), and cortical thickness (C.Th) were used for the morphological measurements and analysis. Bone mineral density was divided into tissue BMD (tBMD) and volumetric BMD (vBMD) by microscopic CT analysis.

### Analysis of born turnover markers in serum

Serum levels of tartrate-resistant acid phosphatase (TRAP), c-terminal crosslinked telopeptides of type I collagen (CTX-1), n-terminal propeptide of type I procollagen (PINP), and alkaline phosphatase (ALP) were analyzed using commercially available ELISA kits (Yanjin Biotech Company Limited, Shanghai), and the values were read using a double beam spectrophotometer.

### Three-point bending experiment of femur

The left femur from experimental animals was removed, and the biomechanical properties were evaluated by a three-point bending test on an electronic universal machine (INSTRON 5584). The loading speed is set at 2 mm/min, the lower span is 1 cm, and the sample is loaded at a constant speed until the sample is completely broken. The test parameters include Maximum loading (N), Stiffness (N/mm), Maximum deflection (mm), and Elastic modulus (Gpa).

### Real-time polymerase chain reaction (RT-PCR) analysis

The left tibiae of both groups of mice were dissolved and fully homogenized with an electric homogenizer and further mixed with a ribonucleic acid (RNA) reagent and the RNA was extracted according to the standard procedures [9]. The extracted RNA was detected on the spectrophotometer, and then RNA reverse transcription was performed according to the kit procedure (TaKaRa Company, Japan). RT-PCR was performed, and the mRNA expression of Runx2, Osx, OPG, and RANKL was determined and normalized to the house-keeping gene (β-actin). The PCR analysis was performed under the following conditions (initial denaturation at 95 °C for 15 s, annealing at 64 °C for 30 s, primer extension at 72 °C for 30 s) about 40–45 cycles.

### Statistics

SPSS 22.0 (IBM Corp., Armonk, NY, USA) was used for all statistical comparisons. All data were expressed as means ± standard deviation (SD). *T* test was used to compare the mean of the two samples, and one-way ANOVA with repeated measures was used to identify significant differences between multiple groups. The two-tailed test was used, and *P* < 0.05 indicated significant differences.

## Results

### Pathological changes of liver in mice with NAFLD

**Fig. 1:**
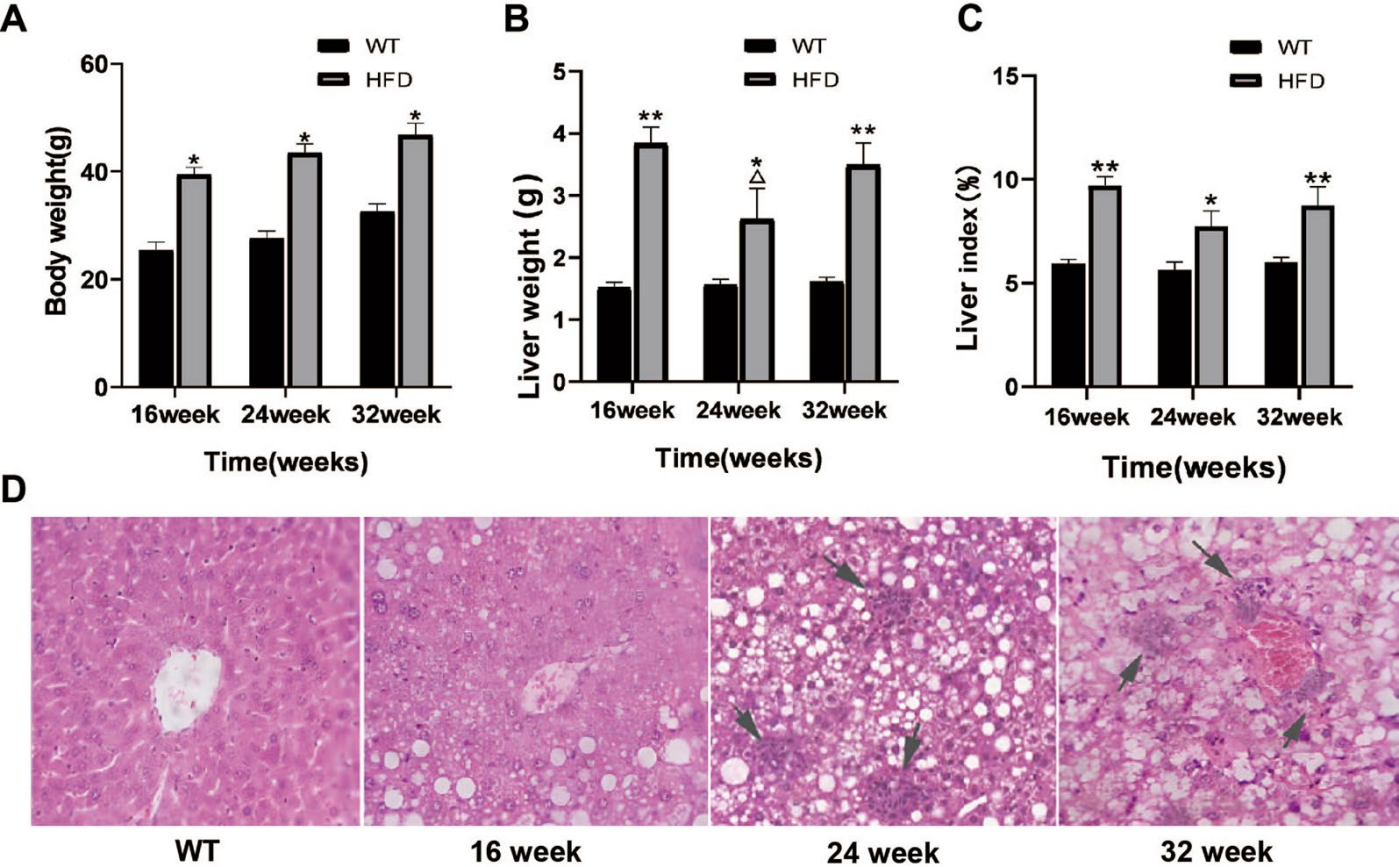
Pathological changes of liver in mice with NAFLD. Comparison of body weight (**A**), liver weight (**B**) and liver index (**C**) in each group of mice. **D** HE staining on liver tissues in mice. *P < 0.05, **P < 0.01 versus WT group; ^△^*P* < 0.05, versus HFD group at 16 week and 32 week

After 16, 24 and 32 weeks of feeding, the body weight of all experimental animals was gradually increased, and the body weight of HFD group was significantly increased compared with that of control group (WT group) (*P* < 0.05, Fig. 1A).There was no significant difference in liver weight at all ages in WT group. However, compared with WT group, liver weight and liver index in HFD group were significantly increased, with the most significant increase at 16 weeks (*P* < 0.01) and decrease at 24 weeks compared with 16 weeks (*P* < 0.05), while increased again at 32 weeks (*P* < 0.01). The liver weight of HFD group at 24 weeks was lower than that at 16 weeks and 32 weeks, showing significant differences (*P* < 0.05, Fig. 1B). The liver index of HFD group was also decreased at 24 weeks, but no significant difference was observed between this time point and others (Fig. 1C). Histological examination of HE-stained liver sections revealed the progression of macrovesicular steatosis at 16 weeks and inflammation cell infiltration at 24 and 32 weeks in the HFD mice compared with the WT mice, in a time-dependent manner (Fig. 1D).

### Serum biochemical analysis of mice with NAFLD

**Fig. 2:**
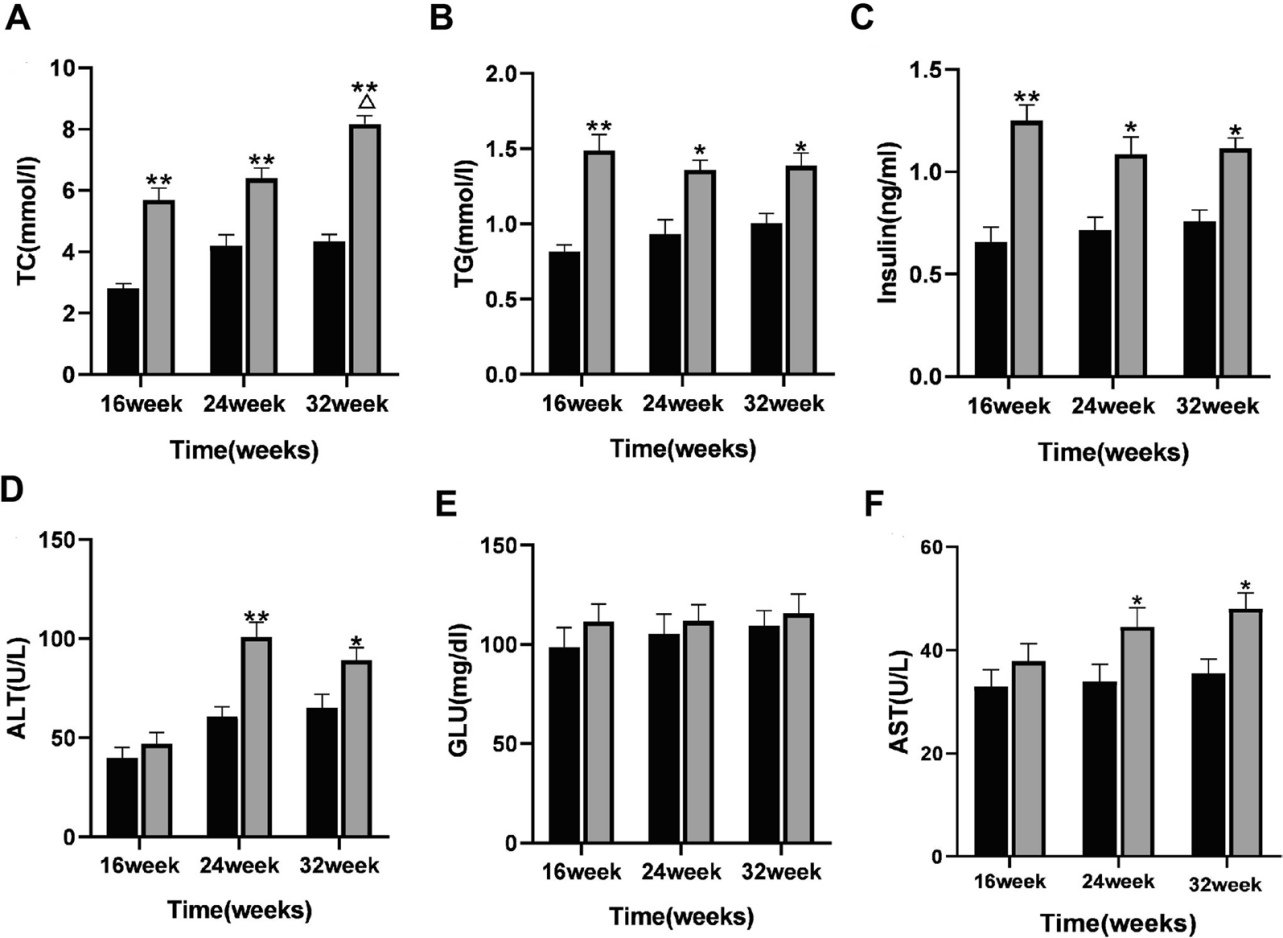
Comparison of serum biochemical detection in mice with NAFLD. Comparison of TC (**A**), TG (**B**), Insulin (**C**), ALT (**D**), GLU (**E**), and AST (**F**) in each group of mice. *P < 0.05, **P < 0.01 versus WT group; ^△^*P* < 0.05 versus 16 week or 24 week group. Scale bar: 50 µm

At 16 weeks, the levels of TG, TC and insulin in HFD group were significantly higher than those in WT group (*P* < 0.01, Fig. 2A–C), which was consistent with NAFLD characteristics, indicating that HFD mouse model was successfully constructed. In contrast, there was no significant difference in AST and ALT levels between the two groups. At 24 weeks and 32 weeks, the levels of TC, TG, ALT, AST and insulin in HFD group were higher than those in WT group, with statistical significance (*P* < 0.05), and the change of ALT was the most significant (*P* < 0.01). At 32 weeks, the TC level in HFD group was significantly higher than that in other age groups. Compared with the control group, GLU level in HFD group was slightly increased at all ages, but the difference was not statistically significant (Fig. 2 D–F).

### Changes of biomechanical properties and bone structures of mice with NAFLD

**Fig. 3:**
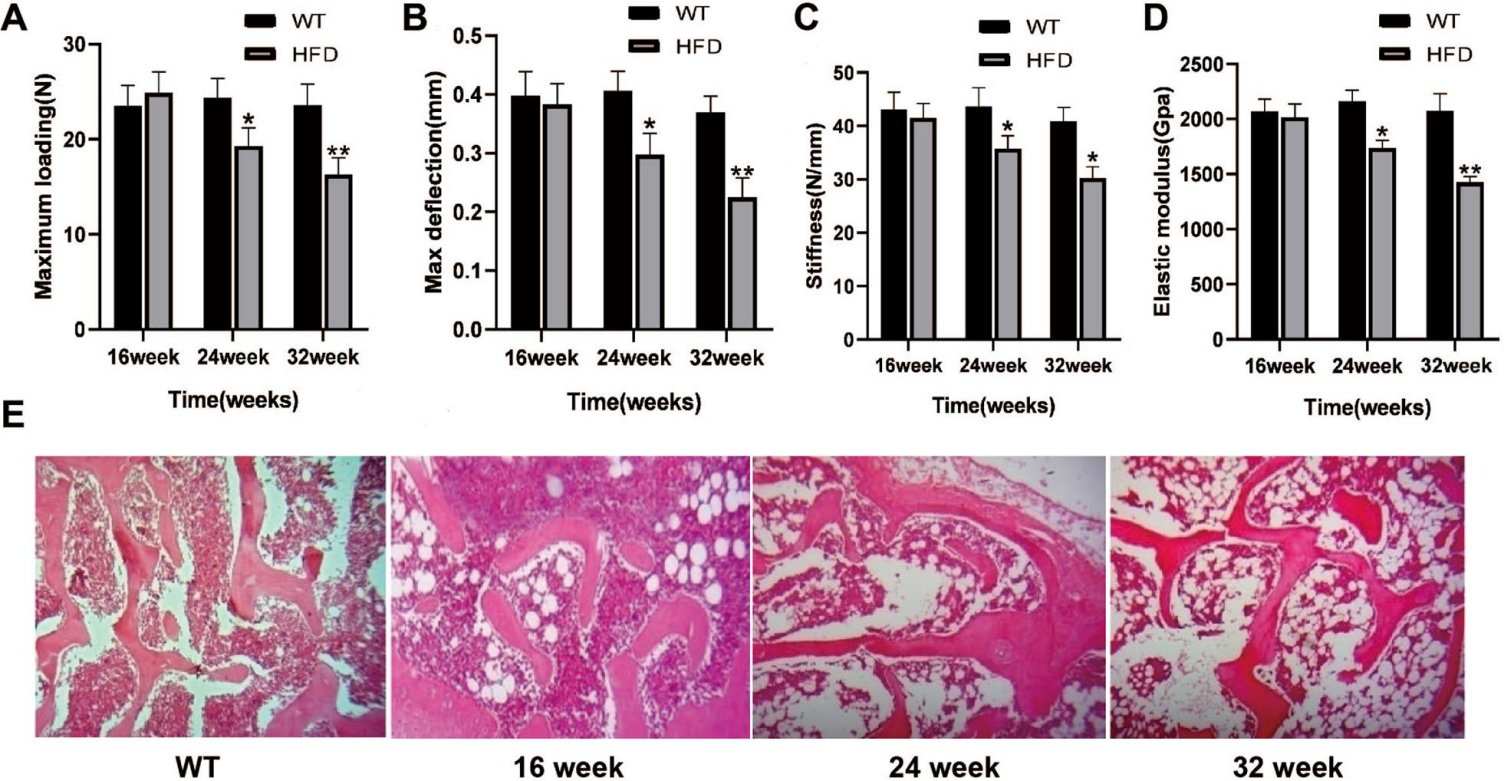
Changes of biomechanical properties and bone structures of mice with NAFLD. Comparison of maximum loading (**A**), max deflection (**B**), stiffness (**C**), and elastic modulus (**D**) in each group of mice. **E** HE staining on tibia tissues in mice. *P < 0.05, **P < 0.01 versus WT group

The results of three-point mechanical test showed that the maximum load was slightly increased and the bending stiffness, maximum deflection and bending elastic modulus were slightly decreased in HFD group at 16 weeks than that of control group, but there was no significant statistical difference, indicating that the femur of mice in HFD group could bear higher weight at 16 weeks, but the toughness was poor. At 24 weeks and 32 weeks, all parameters showed significant decline, and there was a statistical difference than that of control group (*P* < 0.05, Fig. 3A–D), indicating that the bone strength and toughness of mice in HFD group were significantly reduced. In addition, at 16 weeks of HFD group, bone trabeculae was damaged, and the bone damage in HFD group was gradually aggravated in the progression of NAFLD. (Fig. 3E). These data suggest a significant correlation between the progression of NAFLD and the occurrence of osteoporosis.

### Morphology and structural parameters of distal femur

**Fig. 4:**
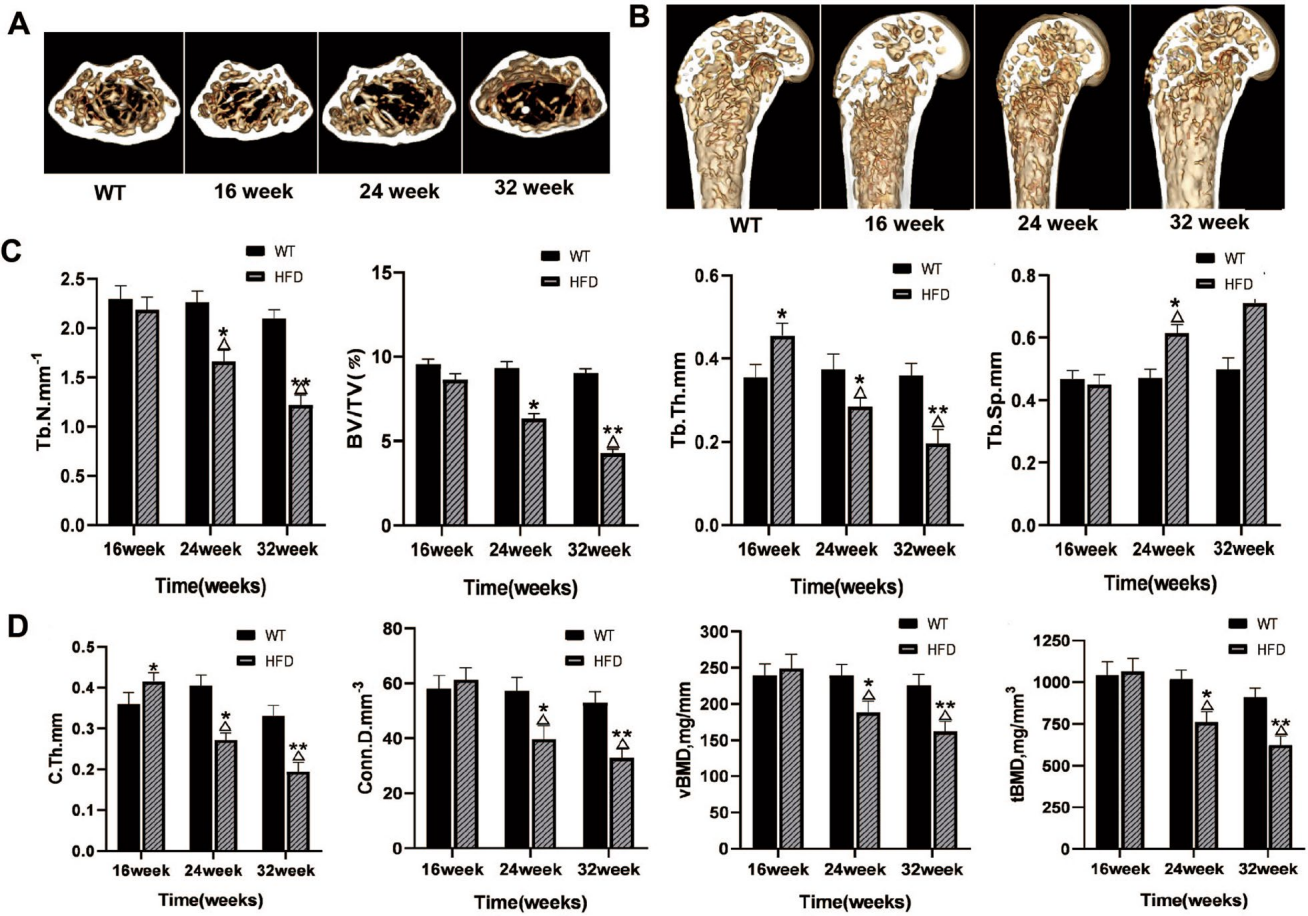
Morphology and structural parameters of the distal femur. **A**, **B** Three-dimensional morphological changes of bone microstructure in distal femurs and trabeculae and cortical thickness in distal femurs of mice in two groups. **C**, **D** Structural features of trabecular bone and cortical bone in the the distal femur of mice at 16, 24 and 32 weeks. *P < 0.05, **P < 0.01 versus WT group; ^△^*P* < 0.05 versus HFD group at 16 weeks

Compared to the WT group, the microstructure of distal femur bone in the HFD group did not change significantly at 16 weeks. From 24 to 32 weeks, the metaphyseal structure of mice in the HFD group had significant changes, and the injuries were aggravated in the progression of disease (Fig. 4A, B). The differences between the HFD and WT group at 16 weeks in the structural parameters or BMD had no significance except Tb.Sp and c.Th. At 24 and 32 weeks, the Tb.N, BV/TV, Tb.Th, c.Th, Conn.D, vBMD, and tBMD in the HFD group was significantly lower than the WT group, whereas the Tb.Sp was remarkably greater in the HFD group (*P* < 0.05 or *P* < 0.01, Fig. 4C, D). In the HFD group, the respective changes of all the metaphyseal structural parameters from 24 to 32 weeks were significant, suggesting the development of osteopenia with the aggravation of NAFLD in HFD group.

### Alterations of TNF-α, IL-6, IGF-1, IGFBP-1, and 25-OH VitD in the serum of mice

**Fig. 5:**
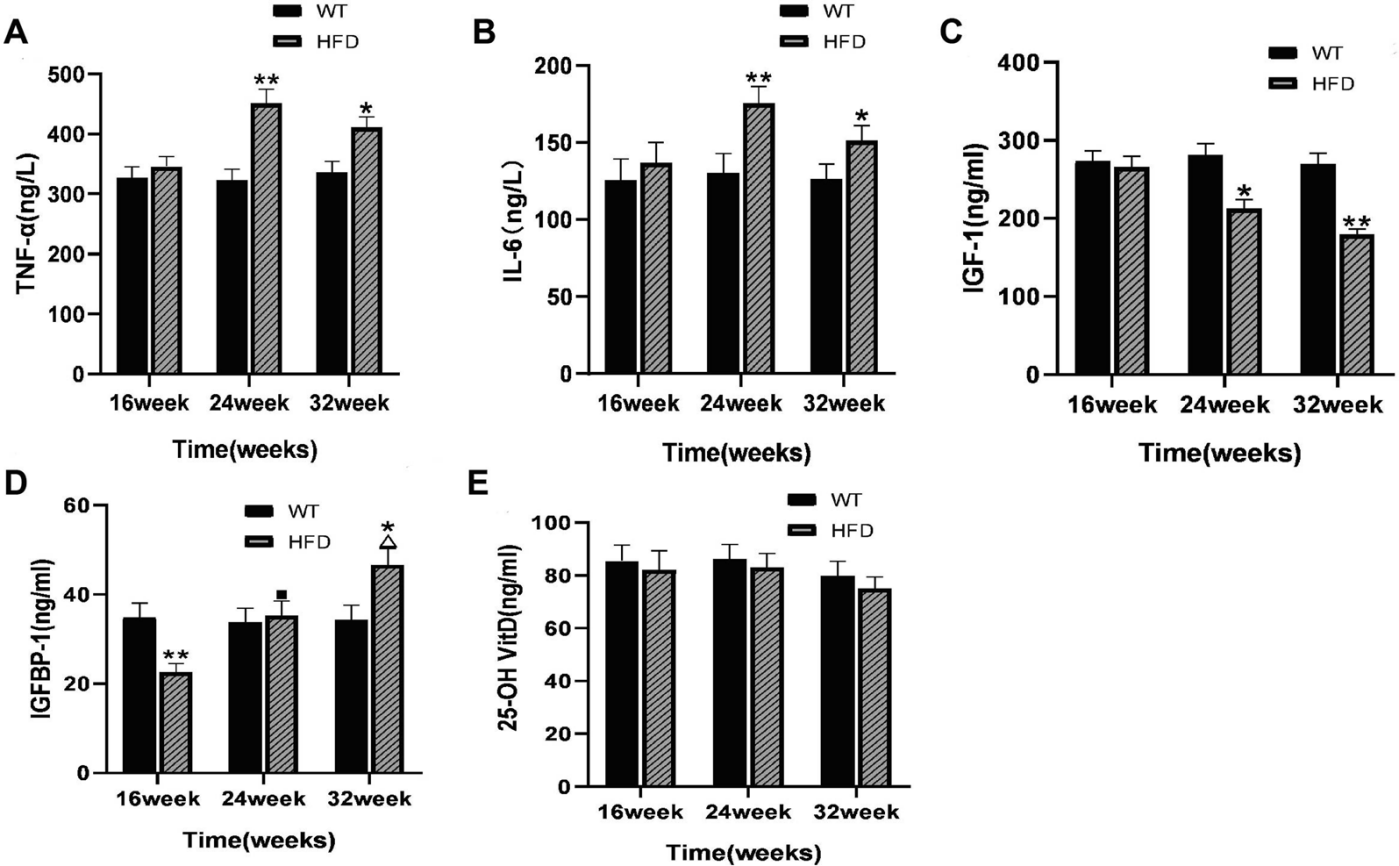
Detection of serum cytokines in mice. The serum levels of TNF-α (**A**), IL-6 (**B**), IGF-1 (**C**), IGFBP-1 (**D**) and 25-OH VitD (**E**) in mice at 16, 24 and 32 weeks were measured by ELISA. *P < 0.05, **P < 0.01 versus WT group; ^■^*P* > 0.05 versus WT group; ^△^*P* < 0.05 versus HFD group at 24 weeks; ^△^*P* < 0.05 versus HFD group at 16 weeks

As shown in Fig. 5A, B, there was no significant difference of TNF-α and IL-6 levels between WT and HFD group at 16 weeks. At 24 weeks, the levels of TNF-α and IL-6 were significantly higher than those in WT group (*P* < 0.01), but the changes decreased at 32 weeks. Compared with WT group, IGF-1 level in HFD group was decreased at 24 weeks (*P* < 0.05), and the difference was more significant at 32 weeks (*P* < 0.01, Fig. 5C). It was found that the IGFBP-1 level was significant lower in HFD group than that in WT group at 16 weeks (*P* < 0.05, Fig. 5D), but no significant difference was observed between the two groups at 24 weeks (*P* > 0.05). At 32 weeks, the IGFBP-1 level increased further in the HFD group, which was significantly higher than that of 24 weeks (Fig. 5D). The differences between the HFD group and WT group on 25-OH VitD level was not significant at any time point (*P* > 0.05, Fig. 5E).

### Changes of serum bone turnover markers in mice

**Fig. 6:**
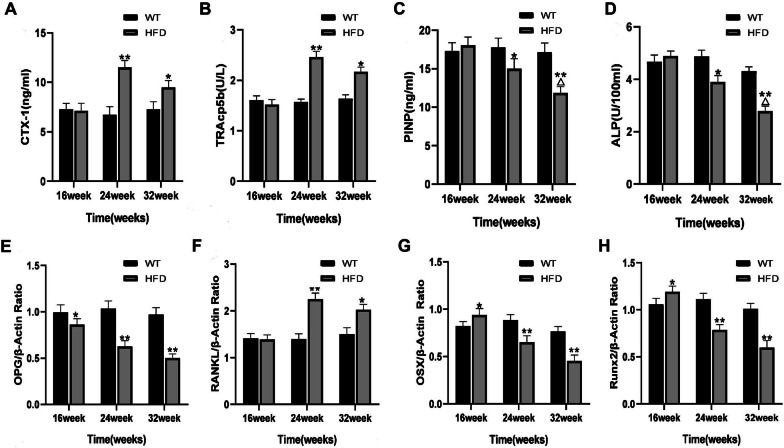
Changes of serum bone turnover markers in mice. **A**–**D** The serum levels of CTX-1, TRACP5b, PINP and ALP in mice of each groups at 16, 24 and 32 weeks were evaluated by ELISA. **E**–**H** The mRNA expression levels of Runx2, Osx, OPG, and RANKL in tibiae of each groups at 16, 24 and 32 weeks were detected by RT-PCR. *P < 0.05, **P < 0.01 versus WT group; ^△^*P* < 0.05 versus HFD group at 24 weeks

To evaluate the bone metabolism of HFD mice, CTX-1, TRACP5b, PINP and ALP in serum of mice were detected by ELISA assay. Compared to the mice in WT group, the levels of PINP and ALP in HFD group were increased at 16 weeks, but the difference was not statistically significant, indicating that osteogenic function of HFD mice was enhanced at 16 weeks, which might be related to the increased body load caused by weight increase. Compared with WT group, serum TRACP5b and CTX-1 contents of HFD mice were significantly increased at 24 weeks (*P* < 0.01) and decreased at 32 weeks (*P* < 0.05) compared with 24 weeks, indicating that osteoclast function was strongest at 24 weeks and decreased at 32 weeks (Fig. 6A, B). The contents of PINP and ALP decreased significantly at 24 weeks (*P* < 0.05) and decreased more significantly at 32 weeks (*P* < 0.01), indicating that with the progression of HFD mice, osteoblastic function was gradually weakened and osteoporosis appeared (Fig. 6C, D).

At 16 weeks, the OPG mRNA level in mouse tibiae of HFD group was decreased, and the mRNA expressions of OSX and Runx2 were increased, while the RANKL mRNA level did not change when compared to the WT group (*P* < 0.05, Fig. 6E–H), suggesting that osteoblast function of mice in HFD group was enhanced, which was considered to be associated with the increase in body weight load. From 24 to 32 weeks, the mRNA levels of OPG, OSX and Runx2 in tibiae of HFD group were decreased in comparison to the WT group, while the RANKL mRNA level showed opposite results (*P* < 0.01, Fig. 6E–H), indicating that osteoblast function was gradually weakened with the aggravation of NAFLD.

## Discussion

Nowadays, growing evidence indicated that NALFD had a close relationship between NAFLD and bone health. However, to date, it remains highly controversial about the relationship between NAFLD and bone health [10]. A HFD containing 60% energy from fat induced NAFLD-resulted metabolic changes in C57BL/6 mice, which can tightly represent human metabolic syndrome, including dyslipidemia, inflammation, and obesity [11]. Li et al. [12] found that the liver lipid content peaked at 16 weeks, significantly promoted the level of pro-inflammatory cytokines at 24 weeks, and fibrosis increased subsequently in some mice at 32 weeks. Studies have found that the prevalence of NAFLD in women increases significantly with age, and the moderate or severe but not mild NAFLD is independently associated with osteoporosis, indicating that the severity of NAFLD in women may be associated with osteoporosis [13, 14]. Based on the above studies, we investigated the serum biochemistry, cytokines and bone microstructure of female mice with NAFLD at 16, 24 and 32 weeks. The Changes of GLU levels could rule out bone loss caused by diabetes.

Histopathological data showed that the bone tissue destruction occurred and gradually worsened with the aggravation of NAFLD in HFD mice. At 16 weeks, the BMD of HFD mice was increased, which was thought to be related to the increase in body load caused by weight increase. Overweight is associated with bone mass to a certain extent, because the increase in weight and fat during exercise makes bones bear greater force, promoting the increase in bone density [6]. However, the bending stiffness and elastic modulus of the bone were decreased in HFD group, indicating that the bone quality was not high and the toughness was poor. At 24 weeks and 32 weeks, the distal femur of mice in HFD group showed significant changes, suggesting that the osteoporosis gradually appeared in HFD group mice with the aggravating of NAFLD.

The second stage of NAFLD is NASH, and insulin resistance is the main pathophysiological change in this stage. Insulin resistance can cause adipose tissue to secrete leptin, IL-6, TNF-α and other adipocytokines, and interact with these factors to cause the ‘second hit’ [15], in which TNF-α plays a key role in this stage [16]. The increase in serum TNF-α level directly stimulates the maturation and activation of osteoclasts and induces IL-6 release from mature osteoclasts. Importantly, IL-6 facilitates the bone resorption and differentiation of osteoclast precursors [17]. TNF-α and IL-6 jointly promote the formation of osteoclasts and bone resorption [18]. In diet-induced NAFLD rodents, RANKL is upregulated in both circulation and liver [19], and blocking RANKL signaling in liver improves liver insulin resistance in HFD mice, suggesting that RANKL plays an important role in the pathogenesis of NAFLD [20]. In bone, RANKL binds to RANK on the surface of osteoclast precursors to promote osteoclast generation and bone resorption, while OPG, a trick receptor, inhibits the intercellular merger of RANKL and RANK, resulting in reduced activity, proliferation and survival of osteoclasts [21, 22]. We found that the serum levels of TNF-α and IL-6 in NAFLD mice increased significantly at 24 weeks, while the level of OPG mRNA in bone tissue decreased significantly and the level of RANKL mRNA increased significantly. Therefore, it can be seen that TNF-α and IL-6 may affect osteoclast function and lead to osteoporosis through the OPG/RANK/RANKL axis. However, the levels of TNF-α and IL-6 did not increased more at 32 weeks than that at 24 weeks. Differently, the changes in metaphyseal structural parameters and BMD were more evident at 32 weeks. Therefore, we considered that there might be other factors that influence bone structure changes in the progressive stages of NAFLD.

Previous evidence has demonstrated that patients with NAFLD have lower IGF-1 level than healthy controls and show a negative correlation between histological severity of NAFLD and IGF-1 level [23]. In 6- and 19-month-old mice, IGF-1 from liver has been shown to be necessary for maintaining cortical bone mass, and the lack of endocrine IGF-1 leads to elevated cortical porosity [24]. Nevertheless, the main principle of IGF-1 affecting bone structure in NAFLD has not been completely understood. We found that serum IGF-1 levels decreased at 24 weeks and were more pronounced at 32 weeks, along with significant decreases in Runx2 mRNA and OSX mRNA levels, which were consistent with BMD changes.

Runx2 is specifically elevated in hepatic stellate cells (mHSC) of NAFLD mice and accelerates the evolution of NAFLD to NASH by inducing macrophage migration in vitro [19]. Meanwhile, Runx2 plays a decisive role in the osteoblastic differentiation from bone marrow mesenchymal stem cells [25]. IGF-1 can activate the MAPK pathway, up-regulate Runx2 mRNA expression, increase the activity of Runx2 [26], and has the ability to promote osteoblast proliferation, differentiation and coupling matrix mineralization [27]. Osx (Osterix) is considered as a downstream target of Runx2, promoting the differentiation of mesenchymal progenitor cells and initiating osteogenesis during osteogenesis, while Osx promotes the maturation of functional osteoblasts [28]. In addition to up-regulating Runx2, IGF-1 also significantly affects the level of OSX and promotes the expression of osteoblast marker genes [29]. It can be seen that the change of IGF-1 signaling pathway affects the expression of Runx2 and OSX and plays an important role in the liver fibrosis stage of NAFLD. However, IGF-1 secreted from the liver is reported to exert a little influence on the growth of cortical periosteal bone and adult axial skeletal, and it is not needed for maintaining the growth of trabecular bone of adult mice [30].

Interestingly, we found that the IGFBP-1 level was significantly increased at 32 weeks. IGFBP-1 is mainly generated by liver cells and is a soluble binding proteins (6 types in total) that controls the bioavailability of IGF-1 via specifically binding to IGF-1 [31]. A pilot study found that there was a close relationship between serum IGF-1 and IGFBP-1 levels and advancement of liver fibrosis of NAFLD [32]. The blood IGFBP-1 was capable of crossing the capillary barrier to exert direct function from cellular perspective [33]. It means that high IGFBP-1 level in the serum indicates high level of IGFBP-1 in the bone [34]. IGFBP-1 binds to integrin-β on the precursor membrane of osteoclasts and enhances RANKL-mediated osteoclast activation and proliferation, leading to bone loss [35]. Therefore, the bone microstructural and BMD changes in NAFLD during the progressive stage were related to IGF-1 and IGFBP-1 changes.

It was reported that diet supplemented vitamin D could improve its own status and could also alleviate disease parameters such as inflammatory, metabolic, and chemical parameters in NAFLD patients [36]. Although low vitamin D level in serum is related to fibrosis and insulin resistance in hepatocytes, while it is not related to bone health [37]. Therefore, it is not clear whether vitamin D affects bone structure in NAFLD.

## Conclusion

To be concluded, this study explored the changes of microstructure of bone over time by micro-CT in a mouse model of NAFLD induced by a high-fat diet. It was found that: (1) Bone loss and the alternations of bone microstructure and BMD occurred in parallel with the progression of NAFLD in HFD-fed mice; (2) Bone structural parameters had no obvious alternations during the ‘first hit’ stage of NAFLD (at 16 weeks). Changes of the inflammatory cytokines TNF-α and IL-6 can induce enhanced osteoclast function through the OPG/RANK/RANKL signaling pathway and may be critical for changes in bone microstructure and BMD during the “second hit” phase of NAFLD (at 24 weeks). Bone microstructural and BMD changes during the progressive stage in NAFLD were related to IGF-1 and IGFBP-1 levels, which reduces the mRNA expressions of Runx and OSX, resulting in decreased osteoblast function (at 32 weeks). As one of the most common chronic liver disorders, the etiology of NAFLD has been intensively studied in patients with increased bone loss regardless of age or gender. Further studies are needed to investigate how liver diseases influence bone metabolism, and our study may bring a new insight to defeat the two intractable diseases including osteoporosis and NAFLD.

### Limitations

This experimental study has only been verified at the animal level, and further exploration at the cellular level will be carried out in the next step.

## Abbreviations

NAFLD: Nonalcoholic fatty liver disease
NASH: Nonalcoholic steatohepatitis
HFD: High-fat diet
WT: Wild-type
micro-CT: Micro-computed tomography
TNF-α: Cytokines tumor necrosis factor-α
IL-6: Interleukin-6
IGF-1: Insulin-like growth factor-1
IGFBP-1: Insulin-like growth factor binding protein-1
ALT: Alanine transaminase
GLU: Serum glucose
AST: Aspartate aminotransferase
TC: Total cholesterol
TG: Triglycerides
IR: Insulin resistance
Tb.Sp: Rabecular separation
BV/TV: Bone volume ratio
Tb.Th: Trabecular thickness
Tb.N: Trabecular number
Conn.D: Connectivity density
C.Th: Cortical thickness
BMD: Bone mineral density
tBMD: Tissue BMD
vBMD: Volumetric BMD
ELISA: Enzyme-linked immunosorbent assay
25(OH)D: 25-Hydroxyvitamin D
OPG: Osteoprotegerin
RANK: Receptor activator of nuclear factor kappa-B
RANKL: Receptor activator of nuclear factor kappa-bligant
Runx2: Runt-related transcription factor 2
Osx: Osterix
CTX-I: C-terminal crosslinked telopeptides of type I collagen
TRAP: Tartrate resistant acid phosphatase
PINP: N-terminal propeptide of type I procollagen
ALP: Alkaline phosphatase
